# Type I error rates of multi-arm multi-stage clinical trials: strong control and impact of intermediate outcomes

**DOI:** 10.1186/s13063-016-1382-5

**Published:** 2016-07-02

**Authors:** Daniel J. Bratton, Mahesh K. B. Parmar, Patrick P. J. Phillips, Babak Choodari-Oskooei

**Affiliations:** MRC Clinical Trials Unit at UCL, 125 Kingsway, London, WC2B 6NH UK

**Keywords:** Multi-arm, Multi-stage, False positive rate, Familywise error rate, MAMS

## Abstract

**Background:**

The multi-arm multi-stage (MAMS) design described by Royston et al. [Stat Med. 2003;22(14):2239–56 and Trials. 2011;12:81] can accelerate treatment evaluation by comparing multiple treatments with a control in a single trial and stopping recruitment to arms not showing sufficient promise during the course of the study. To increase efficiency further, interim assessments can be based on an intermediate outcome (*I*) that is observed earlier than the definitive outcome (*D*) of the study. Two measures of type I error rate are often of interest in a MAMS trial. Pairwise type I error rate (PWER) is the probability of recommending an ineffective treatment at the end of the study regardless of other experimental arms in the trial. Familywise type I error rate (FWER) is the probability of recommending at least one ineffective treatment and is often of greater interest in a study with more than one experimental arm.

**Methods:**

We demonstrate how to calculate the PWER and FWER when the *I* and *D* outcomes in a MAMS design differ. We explore how each measure varies with respect to the underlying treatment effect on *I* and show how to control the type I error rate under any scenario. We conclude by applying the methods to estimate the maximum type I error rate of an ongoing MAMS study and show how the design might have looked had it controlled the FWER under any scenario.

**Results:**

The PWER and FWER converge to their maximum values as the effectiveness of the experimental arms on *I* increases. We show that both measures can be controlled under any scenario by setting the pairwise significance level in the final stage of the study to the target level. In an example, controlling the FWER is shown to increase considerably the size of the trial although it remains substantially more efficient than evaluating each new treatment in separate trials.

**Conclusions:**

The proposed methods allow the PWER and FWER to be controlled in various MAMS designs, potentially increasing the uptake of the MAMS design in practice. The methods are also applicable in cases where the *I* and *D* outcomes are identical.

## Background

The multi-arm multi-stage (MAMS) clinical trial design described by Royston et al. [1, 2] for time-to-event outcomes and by Bratton et al. [3] for binary outcomes is a relatively simple and effective framework for accelerating the evaluation of new treatments. The design has already been successfully implemented in cancer [4] and is starting to be used in other areas such as tuberculosis [5].

In this particular family of MAMS designs, multiple experimental arms are compared to a common control at a series of interim analyses on an appropriate intermediate outcome (*I*) that is on the causal pathway to the definitive primary outcome of the study (*D*). In cancer, a common choice of *D* is overall survival with failure-free survival (a composite of progression-free and overall survival) used for *I* [6]. Alternatively, if a suitable *I* outcome is unavailable then *D* itself or, in some cases, *D* observed at an earlier time point could be used [7]. At each interim analysis, recruitment is stopped to experimental arms that fail to show a predetermined minimum level of benefit over the control on *I*. Recruitment continues to the next stage of the study to all remaining experimental arms and the control. Experimental arms that pass all interim analyses continue to the final stage of the study at the end of which they are compared to the control on *D*.

Two useful measures of type I error rate in a MAMS trial are the pairwise (PWER) and familywise (FWER) type I error rates. The PWER is the probability of incorrectly rejecting the null hypothesis for *D* for a particular experimental arm at the end of the study regardless of other experimental arms in the study. In contrast, the FWER is the probability of incorrectly rejecting the null hypothesis for *D* for at least one experimental arm in a multi-arm study and gives the type I error rate for the trial as a whole. Royston et al. [2] provide a calculation for the PWER; however, it is made under the assumption that the null hypotheses for *I* and *D* for a particular experimental arm are true. In practice, a treatment that is ineffective on *D* may have an effect on *I* different from that under the null hypothesis and we show how this affects the PWER. In particular, the PWER can often be higher than the value calculated by the method of Royston et al. [2] and so we show how to determine and control its maximum value.

In a MAMS trial with more than one experimental arm, controlling the FWER rather than the PWER might be more appropriate particularly if the trial is confirmatory [8]. A calculation of the FWER using a simulation of trial-level data has previously been described in [9] and we use this to show how the FWER can vary for different underlying treatment effects on *I*. We determine the scenario under which the FWER is maximised and thus describe how it may be controlled in the strong sense, that is, for any set of underlying treatment effects on *I* and *D*. In an example, we use the methodology to estimate the maximum PWER and FWER of the original design of the STAMPEDE (Systemic Therapy in Advancing or Metastatic Prostate Cancer: Evaluation of Drug Efficacy) trial in prostate cancer [6] and show how the trial design may have looked had the FWER been controlled in the strong sense at some conventional level.

## Methods

### The MAMS design

Suppose *K* experimental arms are to be compared to a common control over a maximum of *J* stages. In the first *J*−1 stages, experimental arms are compared to the control on an intermediate outcome, *I*, the requirements of which have been described previously [2]. Experimental arms that pass all *J*−1 interim analyses are then compared to the control on *D* at the end of stage *J*. It is also possible for the *I* and *D* outcomes to be the same. For example, a phase II trial is unlikely to consider the efficacy of a treatment on a long-term endpoint that would normally form the *D* outcome in a phase III study (e.g. overall survival) but instead focus only on a single short-term endpoint throughout the study, which could be an indicator for long-term efficacy (e.g. failure-free survival).

Denote by *θ*_*jk*_ the underlying effect of experimental arm *k* relative to control on the outcome in stage *j* ($j=1,\dots,J$; $k=1,\dots,K$). Without loss of generality, assume that a negative value of *θ*_*jk*_ indicates a beneficial effect for arm *k*. Note that a MAMS design currently requires the same null and alternative hypotheses to be used for all arms in the trial, thus allowing each arm to be assessed simultaneously against the control at each interim analysis [3]. Therefore, the null ($H^{0}_{jk}$) and alternative ($H_{jk}^{1}$) hypotheses for *θ*_*jk*_ can be written

$$ \begin{aligned} H_{jk}^{0}&: \theta_{jk} \geq {\theta_{j}^{0}}, \\ H_{jk}^{1}&: \theta_{jk} < {\theta_{j}^{0}}, \end{aligned} \qquad j=1,\dots,J; k=1,\dots,K $$

for some pre-specified null effects ${\theta _{j}^{0}}$. If *I*≠*D* then ${\theta _{j}^{0}}$ is the assumed null value for the effect on *D* and will be denoted by ${\theta _{D}^{0}}$. Likewise, the null effect for the interim stages (*j*<*J*) will be denoted by ${\theta _{I}^{0}}$. If *I*=*D* then ${\theta _{j}^{0}}={\theta _{D}^{0}}$ for all *j*. In practice, ${\theta _{I}^{0}}$ and ${\theta _{D}^{0}}$ are commonly taken to be 0 to represent no difference [e.g. for log hazard ratios (HRs)]. We will also apply similar notation to the underlying treatment effects for each experimental arm: when *I*=*D*, *θ*_*jk*_=*θ*_*Dk*_ for all *j*, while in *I*≠*D* designs, *θ*_*jk*_=*θ*_*Ik*_ for all $j=1,\dots,J-1$ and *θ*_*jk*_=*θ*_*Dk*_ for *j*=*J*. When *K*=1, we will drop the subscript *k*.

The current procedure for designing a MAMS trial is as follows [2]:

- Choose the number of experimental arms, *K*, and stages, *J*, in the trial.
- Define the null values ${\theta _{D}^{0}}$ and, if applicable, ${\theta _{I}^{0}}$ for the effects on the *D* and *I* outcomes, respectively, and specify any corresponding nuisance parameters (e.g. control event rates for binary outcomes, variances for continuous outcomes etc.).
- Choose the allocation ratio *A*, that is the number of patients to allocate to each experimental arm for every patient allocated to the control. *A*=1 represents equal allocation while *A*<1 means that fewer patients will be allocated to each experimental arm than the control.
- For each stage, choose the one-sided significance level, *α*_*j*_, and power, *ω*_*j*_, for all pairwise comparisons in that stage ($j=1\dots,J$). Rough guidelines for choosing *α*_*j*_ and *ω*_*j*_ are described in [2].
- Choose the minimum target differences ${\theta _{I}^{1}}$ and ${\theta _{D}^{1}}$ that one would like to detect on the *I* and *D* outcomes, respectively.
- Calculate the required sample size (or number of events for time-to-event outcomes), timing of each interim analysis and the overall type I error rate (see below) and power. Dedicated software is available in Stata for designing MAMS trials with time-to-event outcomes (nstage) [9, 10] and binary outcomes (nstagebin).

The analysis at the end of each stage occurs when the required sample size in the control arm has completed follow-up or, for time-to-event outcomes, when the required number of events has been observed in the control arm. At each interim analysis (end of stages $1,\dots,J-1$), recruitment is stopped to all experimental arms with observed treatment effects on *I* that are statistically non-significant at level *α*_*j*_, while recruitment to other arms continues into the next stage of the study. Experimental arms that reach the end of the final stage of the study are compared to the control on *D* at level *α*_*J*_ and recruitment to the trial is terminated.

### Pairwise type I error rate

The PWER is the probability of wrongly rejecting the null hypothesis for *D*, ${H_{D}^{0}}$, for a particular experimental arm. Since ${H_{D}^{0}}$ can only be rejected at the end of the final stage of a study, a type I error may only be made at that point (note that this MAMS design can be easily amended to accommodate stopping rules for extreme efficacy on *D*, which will have a negligible impact on the PWER [6]). Furthermore, a type I error cannot be made on the *I* outcome since this is not the primary outcome of the study. For a MAMS trial with *J* stages, Royston et al. [2] state that the PWER is given by

1 $$  \alpha = \Phi_{J}(z_{\alpha_{1}},\dots,z_{\alpha_{J}};R),  $$

where *Φ*_*J*_ is the *J*-dimensional normal distribution function with correlation matrix *R*. The (*j*,*k*)th entry of *R* is the correlation between the treatment effects in stages *j* and *k* under the null hypotheses of *I* and *D*. Calculation of these correlations is described in [2] for time-to-event outcomes, in [3] for binary outcomes and in [11] for a single normally distributed outcome. The overall pairwise power is calculated in a similar manner, replacing the stagewise significance levels (*α*_*j*_) in Eq. 1 with the corresponding stagewise powers (*ω*_*j*_).

#### Influence of an underlying effect on *I* on the PWER

When *I*≠*D*, the calculation of *α* described in [2] is made under the assumption that ${H_{D}^{0}}$ and the null hypothesis for *I*, ${H_{I}^{0}}$, are true. However, in practice it is possible for an experimental arm, to have a beneficial effect on *I* and yet remain ineffective on *D*. Rejecting ${H_{D}^{0}}$ at the end of the study would still constitute a type I error, yet the experimental arm will have a higher chance of reaching that point due to its effectiveness on *I* (i.e. it is more likely to pass the interim stages). Consequently, the PWER for such an arm will be higher than the value calculated in Eq. 1.

**Fig. 1:**
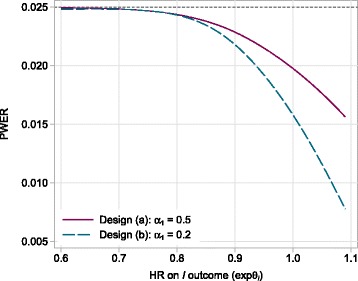
Effect of *θ*_*I*_ on the PWER. The PWER of two 2-stage *I*≠*D* designs when the null effect on *D* is true and the underlying treatment effect on *I* varies. *θ*_*I*_ is the true log HR on the *I* outcome and *α*_*j*_ is the nominal significance level in the *j*th stage (*j*=1,2). *HR* hazard ratio, *PWER* pairwise type I error rates

If the experimental arm is sufficiently effective on *I* that it would always pass all interim analyses, then the first *J*−1 stages effectively become redundant. Under such a scenario, the PWER for the experimental arm would be maximised and will be equal to the final-stage significance level, *α*_*J*_. To illustrate this, Fig. 1 shows the PWERs of two 2-stage *I*≠*D* trial designs with time-to-event outcomes in which the underlying log HR *θ*_*I*_ varies and *θ*_*D*_=0 (i.e. the underlying HR on *D* is 1). The first-stage significance levels are *α*_1_=0.5 in design (a) and *α*_1_=0.2 in design (b). In both designs, the final-stage significance level is *α*_2_=0.025, an equal allocation ratio is used (*A*=1) and ${\theta _{I}^{0}}=0$. Using Eq. 1 to estimate the PWER under the assumption that the experimental arm is ineffective on *I* gives *α*=0.0201 for design (a) and *α*=0.0165 for (b). To calculate type I error rates for other underlying log HRs on *I*, we simulated trial-level data under each design scenario using the procedure described in [9].

As expected, when *θ*_*I*_=0 (i.e. when $\theta _{I}={\theta _{I}^{0}}$), the PWER for both designs is equal to the corresponding value of *α* (Fig. 1). As the effectiveness of the experimental arm on *I* increases (i.e. as *θ*_*I*_ decreases), the PWER eventually plateaus at a level equal to the final-stage significance level (*α*_2_=0.025) with this value being practically reached even for modest effects on *I*. The increase in the type I error rate is greater for design (b) and this will generally be the case when the difference between *α* and *α*_*J*_ is larger. This occurs when using more stages or smaller significance levels in the intermediate stages.

#### Controlling the PWER

Despite it being highly unlikely for a treatment arm to have such an effect on *I* and *D* that the maximum PWER is achieved (particularly if *I* is appropriately chosen), Fig. 1 shows that the inflation in the PWER above the value calculated in Eq. 1 is large even for arms with modest effects on *I*. To help guard against this possibility, one could choose an *I* outcome that has high sensitivity for *D*, since then if there is no effect on *D* it will be highly likely for there also to be no effect on *I*. However, this will not guarantee strong control of the PWER. Therefore, if strong PWER control is required, we recommend setting *α*_*J*_ equal to the desired maximum value, *α*^∗^, when designing a MAMS trial to ensure that it cannot exceed this value under any circumstance.

When the maximum type I error rate in *I*≠*D* designs is controlled using *α*_*J*_, the stopping boundaries for the interim analyses can be considered non-binding. In other words, recruitment to an experimental arm does not strictly have to be stopped at the *j*th interim analysis if its observed treatment effect is statistically non-significant at level *α*_*j*_. This flexibility is advantageous as it may not be desirable to drop arms that are performing no better than the control on *I* if they are showing promising effects on some other important outcome measures. Recruitment to such arms can, therefore, be continued to the next stage without inflating the maximum PWER, although the number of patients recruited will be higher than if the stopping guidelines were strictly followed.

When *I*=*D*, the PWER depends only on the underlying effect on a single outcome (*D*) and so it can be accurately estimated using Eq. 1. In contrast to the *I*≠*D* case, all stagewise significance levels contribute to this maximum value and so stopping boundaries must be binding (i.e. strictly adhered to) to avoid inflating *α*. If this is likely to be impractical due to the above reasons, then the maximum PWER can instead be controlled in a similar manner to the *I*≠*D* case by setting *α*_*J*_=*α*^∗^ to allow stopping boundaries to be non-binding. Note, however, that this will come at the expense of an increase in the sample size for the final stage of the study due to the use of a smaller significance level in that stage.

### Familywise error rate

When evaluating more than one experimental arm in a single study, the probability of at least one false-positive result, the FWER, will be higher than the PWER [12]. In many multi-arm settings, it may, therefore, be more desirable to control the type I error rate for the trial as a whole at some conventional level rather than for each individual treatment comparison.

In a MAMS design, the FWER can be calculated using a generalisation of a simulation procedure proposed by Wason and Jaki [11] for MAMS trials with a single outcome and equally spaced interim analyses. The procedure works by simulating the joint distribution of the *z*-test statistics for each arm at each stage of the study, accounting for the between-arm and between-stage correlations of the treatment effects. For MAMS designs with *I*=*D*, the maximum FWER occurs under the global null hypothesis (i.e. when ${H_{D}^{0}}$ is true for all experimental arms) [13, 14]. When *I*≠*D*, the FWER is maximised when all experimental arms are sufficiently effective on *I* that they would always pass all interim analyses but are all ineffective on *D*, i.e. when $\theta _{Ik}=-\infty $ and $\theta _{Dk}={\theta _{D}^{0}}$ for all *k* [9]. In this case, the interim stages effectively become redundant and the design reduces to a one-stage trial with the PWER equal to the final-stage significance level, *α*_*J*_ (i.e. the maximum PWER). The maximum FWER can, therefore, be computed more quickly using the Dunnett probability: [15]

2 $$  \text{FWER} = 1-\Phi_{K}(z_{1-\alpha_{J}},\dots,z_{1-\alpha_{J}};C),  $$

where *C* is the *K*×*K* between-arm correlation matrix with off-diagonal entries equal to *A*/(*A*+1).

#### Influence of the underlying effects on *I* on the FWER

To illustrate how quickly the maximum value of the FWER is reached as the true treatment effects on *I* vary, we calculated the FWER for designs (a) and (b) described in the previous section when two experimental arms are compared to the control. In both two-stage designs, we assumed $\theta _{Dk}={\theta _{D}^{0}}$ (i.e. *θ*_*Dk*_=0, *k*=1,2) while the underlying effects on *I* in one or both experimental arms varied. For both designs, the maximum FWER (calculated in nstage using Eq. 2) is 0.045. Note that this maximum value is the same for both designs as they have identical numbers of experimental arms (*K*=2), allocation ratios and final-stage significance levels. Assuming the null hypothesis on *I* holds for both arms (i.e. the log HRs on *I* are 0), then the FWER is estimated using nstage to be 0.0372 and 0.0305 for designs (a) and (b), respectively. In this case, the FWER is lower for design (b) as it uses a lower significance level in the first stage.

**Fig. 2:**
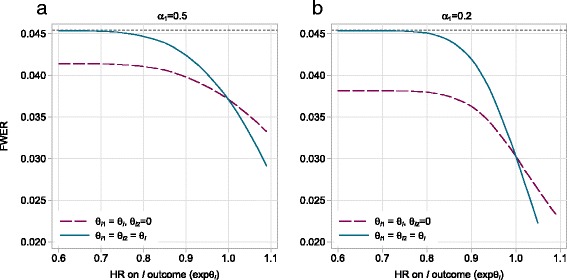
Effect of *θ*_*I*_ on the FWER. The FWER of two 3-arm two-stage *I*≠*D* designs when both experimental arms are ineffective on *D* but the underlying treatment effects on *I* vary in one or both experimental arms. *θ*_*Ik*_ is the underlying log HR of arm *k* on *I*. **a** *α*_1_=0.5. **b** *α*_1_=0.2. *HR* hazard ratio, *FWER* familywise error rate

To calculate the FWER when the underlying effects on *I* in one or both experimental arms vary, we used the simulation procedure described in [9]. The results presented in Fig. 2 show that when both experimental arms are even modestly effective on *I* (e.g. HR=0.8), the maximum FWER is practically reached. The rate of inflation in the FWER as the underlying effects on *I* increase is again greater for design (b), as was the case for the PWER. When only one experimental arm is effective on *I*, the FWER is still substantially higher than under the global null hypothesis on *I*, although only by about half the amount when both arms are effective on *I*.

#### Controlling the FWER

When *I*≠*D*, the FWER as well as the PWER can be controlled in the strong sense using the final-stage significance level alone. To find the value of *α*_*J*_ corresponding to the desired FWER, a search procedure over *α*_*J*_ can be used. For example, to find the required value of *α*_*J*_ that controls the maximum FWER at the one-sided 2.5 % level in designs (a) and (b), we used nstage iteratively to calculate the maximum FWER of the designs using values of *α*_*J*_ between 0.0125 and 0.025 (the minimum and maximum possible values of *α*_*J*_ that can correspond to the maximum FWER) in increments of 0.0001. The final-stage significance level that most closely corresponded to a FWER of 0.025 without exceeding it was 0.0135. Alternatively, the qmvnorm function in R can also be used to compute the required values of *α*_*J*_.

When *I*=*D*, it is more difficult to find designs that control the FWER since a search procedure over all stagewise significance levels is required. Since *I*=*D* designs are also likely to be used in practice, a method for controlling the FWER in the *I*=*D* case is needed and is an area of ongoing research. However, if researchers wish to have the flexibility of non-binding stopping guidelines, then the maximum FWER can be controlled in the same manner as for an *I*≠*D* design and so the methods described above can be applied.

## Results

The STAMPEDE trial in prostate cancer started as a six-arm four-stage trial using the methodology described by Royston et al. [1, 2]. The trial used failure-free survival as *I* and overall survival as *D*. Recruitment began in 2005 and was completed in 2013. The original design of the trial is shown in Table 1. An allocation ratio of *A*=0.5 was used for this design so that one patient was allocated to each experimental arm for every two patients allocated to the control. Because distinct hypotheses were being tested in each of the five experimental arms, the design focus for STAMPEDE was on the pairwise comparisons of each experimental arm against control, with emphasis on the control of the pairwise type I error.

**Table 1:** Design of the six-arm four-stage STAMPEDE trial in prostate cancer

Stage (*j*)	Target	Outcome	One-sided significance	Power (*ω*_*j*_)	Required control
	HR		level (*α*_*j*_)		arm events
1	0.75	FFS	0.500	0.95	113
2	0.75	FFS	0.250	0.95	216
3	0.75	FFS	0.100	0.95	334
4	0.75	OS	0.025	0.90	403
Overall			0.013	0.83	

*FFS* failure-free survival, *HR* hazard ratio, *OS* overall survival

Using Eq. 1, the PWER was estimated to be 0.013. However, as explained above, the maximum PWER is actually equal to the final-stage significance level, *α*_4_=0.025. Using the calculation described in ‘Methods’, the maximum FWER of the original STAMPEDE design was 0.103.

Although the FWER was not controlled in STAMPEDE, below we use the trial in an example to show how strong FWER control can be achieved in a MAMS design with *I*≠*D*. Using a search procedure over *α*_4_ in nstage, similar to that used above for the two-stage designs, we found that final-stage significance levels of *α*_4_=0.0054 and *α*_4_=0.0113 would have been required to control the FWER at 2.5 % and 5 %, respectively. Stata code for determining the final-stage significance level for a FWER of 2.5 % is shown in the Appendix.

Consequently, this would have increased the required number of *D* events on the control arm in the final stage from 403 to 558 and 485, respectively (as estimated by nstage) and may, therefore, have led to a prolonged trial should any experimental arm reach the final stage. Thus, investigators designing and conducting a trial should consider carefully the necessity of controlling the FWER in their trial, and whether it is achievable from a practical point of view.

## Discussion

The MAMS design is an effective and relatively simple approach for accelerating the evaluation of multiple new treatments. It works by simultaneously assessing experimental arms against a common control in a single trial, stopping recruitment to poorly performing arms during the trial, and allowing interim assessments to be based on an outcome that is observed earlier than the primary outcome of the study. In this article, we described how the type I error rate for each individual experimental arm and for the trial as a whole can be determined and controlled in *I*≠*D* designs and *I*=*D* designs with non-binding stopping guidelines. We also investigated the impact of the underlying treatment effect on the type I error rate in *I*≠*D* designs and showed that it is possible for the PWER to be higher than previously thought, with the maximum value being equal to the final-stage significance level of the trial, *α*_*J*_. Similarly, for *I*≠*D* designs with more than two arms, the maximum FWER does not depend on the stagewise significance levels prior to the final stage and can be calculated simply by treating the design as a standard one-stage trial with the PWER equal to *α*_*J*_. We found that even for arms with modest effects on *I* but no effect on *D* (a scenario often seen in practice), the type I error rate can approach quite rapidly to these maximum values. Thus, controlling the maximum PWER or FWER should be an important design consideration in any future MAMS trials.

An advantage of controlling the maximum PWER or FWER of the trial by *α*_*J*_ is the increased flexibility of allowing recruitment to poorly performing experimental arms to be continued to the next stage without inflating the type I error rate. This flexibility allows arms showing promising effects on other important outcome measures to be assessed further, albeit at the expense of a larger sample size. Interim stopping guidelines can also be non-binding in *I*=*D* designs if the maximum PWER and FWER are controlled by *α*_*J*_ only. Another benefit is that the FWER calculation is somewhat simplified and is similar to the Dunnett procedure for a one-stage trial [15]. However, *I*=*D* MAMS designs with binding stopping rules may also be used in practice and so a method for controlling their PWER or FWER is required. Alternatively, other approaches to designing MAMS trials with a single normally distributed outcome have been proposed in [11, 13]. Methods for controlling the FWER in these designs are available (e.g. using the mams package in R) and, unlike the MAMS designs we have considered in this paper, stopping guidelines for efficacy such as those in standard group sequential trials (e.g. [16, 17]) can be built into the design. Other approaches are also available for multi-arm trials with strong FWER control where only the most promising treatment is to be selected at an interim analysis based on a combination of both short- and long-term endpoint data [18, 19]. Such designs are, therefore, more suited to situations where the best of several treatments is to be determined, as might often be the case in a pharmaceutical setting.

There is currently much debate over whether the FWER should be controlled in a multi-arm study. It has been argued that FWER control is most appropriate in confirmatory settings [20] and has also been proposed for exploratory studies to limit the chance of evaluating an ineffective treatment in a potentially expensive confirmatory study [8]. However, Hughes [21] argues that adjusting for multiple comparisons should not be a requirement, since no such adjustment would have been made if each experimental arm were evaluated in a separate two-arm study. Freidlin et al. [22] suggest that this argument is only reasonable if each treatment is distinct and a multi-arm trial was used purely for efficiency reasons. If, on the other hand, the experimental arms are closely related (e.g. if they are different doses or schedules of the same drug), then the FWER should be controlled. Despite this guidance, Wason et al. [12] show that many multi-arm confirmatory trials do not correct for multiple testing even if the treatments are closely related. It remains unclear whether the FWER should be controlled in confirmatory trials of several distinct treatments and further guidance from regulators is required [12].

There has recently been much discussion over the adding of arms to an ongoing MAMS design, such as the STAMPEDE trial, which to date has added three new arms since it commenced [8, 23, 24]. The effect of adding new experimental arms is advantageous as it obviates the often lengthy process of initiating a new trial. However, the impact of adding arms on the FWER in the class of MAMS designs discussed here has not yet been fully explored. Therefore, methods for quantifying and, in some cases, controlling the FWER in such a trial are required. In addition, it is not initially clear how much the FWER will be inflated when arms are added only when existing arms are dropped for lack of benefit. A related question is whether a sequentially rejective procedure, such as that described by Proschan et al. [25], could be applied to the MAMS design [26]. Such a procedure relaxes future stopping guidelines if arms are dropped during the course of the trial, so that the power for the remaining comparisons is increased without inflating the FWER. For instance, if a two-stage trial initially has two experimental arms and recruitment to one arm is stopped at the first analysis, then the question is whether a final-stage significance level that is higher than that proposed in the initial design could be used.

## Conclusions

In this paper, we described how to calculate the maximum PWER and FWER of a MAMS design and have presented methods for controlling these measures at some desirable level for *I*≠*D* designs and *I*=*D* designs with non-binding stopping guidelines. The Stata software for designing MAMS trials has been updated accordingly [9].

## Appendix

Below is the Stata code used to determine the final-stage significance level required to strongly control the FWER of the original STAMPEDE design at the one-sided 2.5 % level, as shown in ‘Results’. The code can be easily amended for a user’s own *I*≠*D* MAMS trial. Full details of the nstage Stata program are described in [9].



## Abbreviations

FFS, failure-free survival; FWER, familywise error rate; HR, hazard ratio; MAMS, multi-arm multi-stage; OS, overall survival; PWER, pairwise error rate; STAMPEDE, Systemic Therapy in Advancing or Metastatic Prostate Cancer: Evaluation of Drug Efficacy
